# Hyperuricaemia, gout and allopurinol in the CKD Queensland registry

**DOI:** 10.1007/s40620-020-00937-4

**Published:** 2021-01-13

**Authors:** A. Jeyaruban, W. Hoy, A. Cameron, H. Healy, Z. Wang, J. Zhang, A. Mallett

**Affiliations:** 1 CKD.QLD and NHMRC CKD.CRE, Brisbane, QLD Australia; 2 Faculty of Medicine, The University of Queensland, Herston, QLD Australia; 3 Kidney Health Service, Royal Brisbane and Women’s Hospital, Level 9 Ned Hanlon Building, Butterfield Street, Herston, QLD 4029 Australia

**Keywords:** Hyperuricaemia, Gout, Allopurinol, CKD, ESKD

## Abstract

**Introduction:**

There is scant data on the role of hyperuricaemia, gout and allopurinol treatment in chronic kidney disease (CKD). Therefore, our aim is to investigate the possible associations between hyperuricaemia, gout, prescription of allopurinol and renal outcomes in patients with CKD.

**Methods:**

The retrospective cohort study involved 1123 Royal Brisbane and Women’s Hospital (RBWH) patients, enrolled in the CKD.QLD registry from May 2011 to August 2017. Patients were divided into two uric acid categories, with uric acid ≤ 0.36 mmol/L and > 0.36 mmol/L. Association of delta estimated glomerular filtration rate (eGFR) with gout, allopurinol treatment and hyperuricaemia were analysed.

**Results:**

Patients with an entry urate > 0.36 mmol/L were older, had higher body mass index (BMI) and worse baseline kidney function. Proportion of patients with gout, hyperuricaemia and allopurinol treatment increased with advanced CKD stages. Age-adjusted analysis revealed a significant association between serum urate level and delta eGFR, with no significant association between gout, treatment with allopurinol and delta eGFR. Furthermore, neither gout nor the prescription of allopurinol had a significant effect on the time to renal death (composite end point of kidney replacement therapy or death).

**Conclusion:**

Hyperuricaemia seemed to be independently associated with faster CKD progression or renal death. This was not observed with gout or prescription of allopurinol. Furthermore, allopurinol was not associated with decreased incidence of cardiovascular events. These data suggest that hyperuricaemia is likely the effect and not the cause of CKD or CKD progression.

**Graphic abstract:**

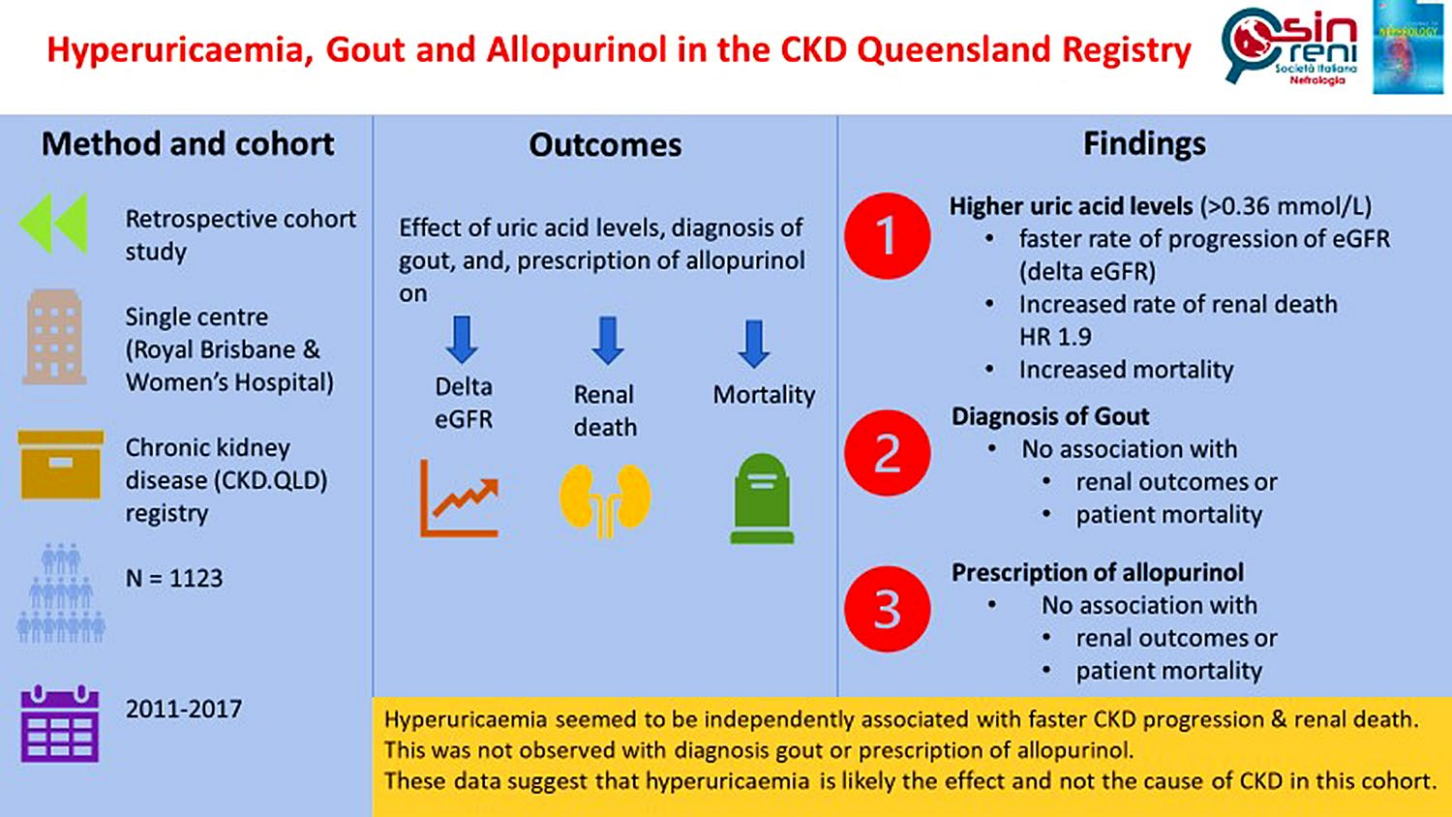

## Introduction

Chronic Kidney Disease (CKD) is a global health issue [1]. The progression of CKD leads to end stage kidney disease (ESKD), requiring kidney replacement therapy (KRT) and is associated with excess mortality [2], cardiovascular events [3] and hospitalisations [4]. Increased serum uric acid levels have been found to independently predict the development of CKD [5]. Some epidemiological studies have reported that hyperuricaemia is a possible risk factor for the development and further deterioration of kidney disease [6, 7]. Furthermore, animal experimental studies found that rising serum uric acid levels induce an oxidative stress response and endothelial dysfunction. This could lead to the development of systemic and glomerular hypertension which could subsequently elevate renal vascular resistance and reduce renal blood flow [8–10]. However, other investigators found no (observed) association between serum uric acid levels and progression of CKD [11].

The association between the diagnosis of gout and the prescription of urate lowering therapy on renal outcomes is not well characterised. The effects of one of these drugs, allopurinol, include improved endothelial function and arterial wave reflection [12]. Small pilot studies reported mixed results regarding the relationship between allopurinol prescription and renal outcomes. Siu and colleagues reported an association between allopurinol and decreased renal deterioration, whilst Goicoechea and colleagues found no association [13, 14]. Given the physiological impact of allopurinol on vasculature, we hypothesised that allopurinol prescription would slow down the progression of renal deterioration in patients with CKD, and also decrease cardiovascular events (ischaemic heart disease, stroke or peripheral vascular disease) in the CKD population. Moreover, we hypothesised that hyperuricaemia and a diagnosis of gout would be associated with an increased rate of renal deterioration in a CKD cohort. Our aim, therefore, is to evaluate the association between serum uric acid levels, diagnosis of gout, prescription of allopurinol and renal outcomes at one site in the CKD.QLD cohort.

## Methods

### Source of data

CKD.QLD is a state-wide collaborative multidisciplinary research and practice program that was established in 2011 [15]. It involves a CKD registry of all consented patients in the public health system in the state of Queensland, Australia. We included patients enrolled in the CKD.QLD registry from Metro North Hospital and Health Service in Brisbane, Australia. The database captures extensive information on date of birth, gender, medical background, and primary diagnosis for renal disease. Serum uric acid levels on entry were extracted from the state-wide public pathology provider, “Pathology Queensland”, whilst information on the diagnosis of gout and allopurinol prescription was obtained from the integrated medical records.

### Ethics

The study was approved by the following Human research ethics committee; Royal Brisbane and Women’s Hospital, Queensland Health—HREC/15/QRBW/294 and the Medical research ethics committee, University of Queensland—2011000029. The study has been approved with subsequent protocol amendments and extensions.

### Study design

To investigate the association between hyperuricaemia, diagnosis of gout, prescription of allopurinol and renal outcomes, we performed a retrospective cohort study involving 1123 patients from the CKD.QLD registry from the Metro North Hospital and Health Service. The patients were followed from 1st of May, 2011 to 31st of August, 2017. Patients below the age of 18 and those who had been commenced on KRT prior to May 2011 were excluded from this study.

### Statistical analysis

SPSS software was used to analyse the univariate and multivariate association between serum uric acid levels, diagnosis of gout, prescription of allopurinol and renal outcomes. The patient cohort was described as means as well as percentages. The concept of delta estimated glomerular filtration rate (eGFR) was used as the metric of progression of kidney disease. Delta eGFR was calculated by subtracting the latest eGFR (CKD EPI) from the initial eGFR (CKD EPI) and then dividing that by the time in years between the two data points. Chi square analysis between the groups based on presence or absence of hyperuricaemia, diagnosis of gout and prescription of allopurinol were compared to the comorbidities to ascertain an association between these variables. T tests were used to determine the significance of the association between serum uric acid levels, diagnosis of gout, prescription of allopurinol and the delta eGFR. Kaplan Meier analysis with log rank test and Cox proportional hazard modelling were used to determine the association between the above variables and time to renal death. The term renal death was defined as the onset of kidney replacement therapy or death. This was done for two reasons. Firstly, there was a large number of patients who died during the follow up and secondly, deaths are a competitive factor for end stage renal failure. The onset of renal death or patient death was subsequently used as an endpoint. Kaplan Meier analysis was also used to determine the association between the prescription of allopurinol and the time to new cardiovascular events during the follow up time. A *p* value < 0.05 was regarded as statistically significant.

## Results

### Cohort

A total of 1123 patients were analysed. The patients were followed up for a total of 7 years. The median follow-up was 2070 days and the range was 2404 days. The median age of the cohort was 70 years with an interquartile range of 21. There were 546 (49%) females and 577 males (51%). One hundred twenty-six patients (11%) progressed to end stage renal failure requiring KRT. In this cohort, there was a total of 274 deaths. Of these deaths, 41 were attributed to renal failure, 53 to sepsis, 40 to cardiac causes, 40 to cancer, 15 to respiratory causes, 14 to stroke and 60 to others and unknown causes.

### Association between uric acid and progression of kidney disease

**Fig. 1:**
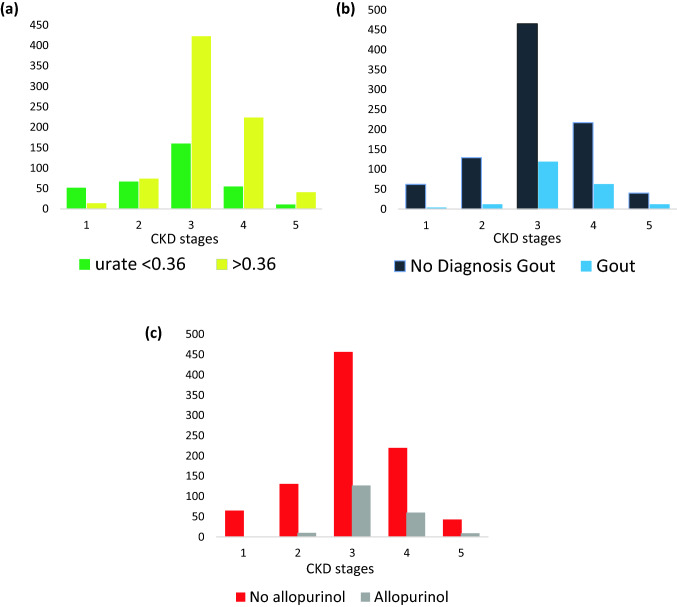
Association between serum uric acid level (**a**), diagnosis of gout (**b**) prescription of allopurinol (**c**) and Chronic kidney disease stages

There were 775 patients with a serum uric acid level greater than 0.36 mmol/L (reference range 0.18–0.36 mmol/L). Their baseline characteristics are summarised in Table 1a and compared with the patient cohort with a serum uric acid level less than or equal to 0.36 mmol/L. There were no significant differences in age and body mass index (BMI) between the two groups. However, the patients with a serum uric acid level greater than 0.36 mol/L had significantly worse kidney function and higher prevalence of hypertension, diabetes mellitus, dyslipidaemia, ischaemic heart disease, stroke and gout. With increasing stages of CKD, the prevalence of patients with a serum uric acid level greater than 0.36 mmol/L increased (Fig. 1a). On bivariate analysis, there was a significant association between hyperuricaemia and delta eGFR, with a mean delta eGFR of 1.68 ml/min/1.73m2/yr (std dev: 8.3) in the cohort with serum uric acid level less than or equal to 0.36 mmol/L compared to 3.1 ml/min/1.73m2/year (std dev: 6.3) in those with serum uric acid level greater than 0.36 mmol/L (p value < 0.05) Table 2a). This was further reiterated in Pearson correlation analysis by utilising serum uric acid levels as a continuous variable. This analysis also showed a significant association between initial serum uric acid levels and delta eGFR. Table 2a summarises the univariate analysis exploring the association between relevant patient factors and delta eGFR.

**Table 1:** (a) Characteristics of patients based on initial serum uric acid levels; (b) Characteristics of patients based on diagnosis of gout; (c) Characteristics of patients based on prescription of allopurinol

(a)			
Baseline features	Urate ≤ 0.36 mmol/L N(%), Median (IQR)	Urate > 0.36 mmol/L N(%), Median (IQR)	p value
Total	346	775	
Age (years)	67 (24)	71.5(18)	0.1
eGFR (mL/min/1.73 m^2^)	47 (38)	35 (22)	< 0.05
Body mass index (kg/m^2^)	28(10)	30(10)	0.1
CKD stage 1	52 (4.6%)	14 (1.2%)	< 0.05
CKD stage 2	67 (6%)	74 (6.6%)	0.6
CKD stage 3	160 (14.2%)	423 (37.7%)	< 0.05
CKD stage 4	55 (4.9%)	224 (19.9%)	< 0.05
CKD stage 5	12 (1.1%)	40 (3.6%)	< 0.05
Hypertension	214 (61.9%)	594 (76.7%)	< 0.05
Diabetes	129 (37.3%)	385 (49.7%)	< 0.05
Dyslipidaemia	124 (35.8%)	351 (45.3%)	< 0.05
Ischaemic heart disease	73 (21.1%)	249 (32.1%)	< 0.05
Gout	43 (12.4%)	160 (20.7%)	< 0.05
Allopurinol treatment	43 (12.4%)	160 (20.7%)	< 0.05
Stroke	21 (6.1%)	94 (12.1%)	< 0.05
Peripheral vascular disease	36 (10.4%)	115 (14.8%)	0.1
Heart failure	19 (5.5%)	66 (8.5%)	0.1

(b)			
	No diagnosis of gout N(%), Median (IQR)	Diagnosis of gout N(%), Median (IQR)	p value
Total	913	210	
Age (years)	70 (22)	71 (15)	< 0.05
eGFR (mL/min/1.73 m^2^)	39 (26)	35 (20)	< 0.05
Body mass index (kg/m^2^)	29 (9)	30 (10)	0.3
CKD stage 1	62(5.5%)	4 (0.4%)	< 0.05
CKD stage 2	129 (11.5%)	12 (1.1%)	< 0.05
CKD stage 3	465 (41.4%)	119 (10.6%)	< 0.05
CKD stage 4	217 (19.3%)	63 (5.6%)	< 0.05
CKD stage 5	40 (3.6%)	12 (1.1%)	< 0.05
Hypertension	663 (72.6%)	170 (81%)	0.01
Diabetes	412 (45.1%)	115 (54.8%)	0.01
Dyslipidaemia	377 (41.3%)	109 (51.9%)	0.05
Ischaemic heart disease	264 (28.9%)	67 (31.9%)	0.4
Allopurinol treatment	95 (10.4%)	111 (52.9%)	< 0.05
Stroke	95 (10.4%)	24 (11.4%)	0.7
Peripheral vascular disease	126 (13.8%)	31 (14.8%)	0.7
Heart failure	63 (6.9%)	23 (11%)	0.06

(c)			
	Prescription of allopurinol N(%), Median (IQR)	No prescription of allopurinol N(%), Median (IQR)	p value
Total	207	916	
Age (years)	72 (16)	70 (23)	< 0.01
eGFR (mL/min/1.73 m^2^)	35 (18)	39 (27)	< 0.01
Urate (mmol/L)	0.45 (0.19)	0.41 (0.15)	< 0.01
Body mass index (kg/m^2^)	30.5 (10)	29 (9)	< 0.01
CKD stage 1	1 (0.1%)	65 (5.8%)	< 0.05
CKD stage 2	10 (0.9%)	131 (11.7%)	< 0.05
CKD stage 3	127 (11.3%)	457 (40.7%)	< 0.05
CKD stage 4	60 (5.3%)	220 (19.6%)	< 0.05
CKD stage 5	43 (3.8%)	9 (0.8%)	< 0.05
Hypertension	173 (83.6%)	660 (72.1%)	< 0.01
Diabetes	111 (53.6%)	416 (45.5%)	< 0.04
Dyslipidaemia	111 (53.6%)	375 (40.9%)	< 0.01
Ischaemic heart disease	78 (37.7%)	253 (27.6%)	< 0.05
Stroke	25 (12.1%)	94 (10.3%)	0.5
Peripheral vascular disease	32 (15.5%)	125 (13.6%)	0.5
Heart failure	26 (12.6%)	60 (6.6%)	< 0.05

**Table 2:** (a) Cox analysis (unadjusted) exploring factors associated with renal outcomes (delta eGFR); (b) Multivariate analysis exploring factors associated with renal outcomes (delta eGFR); (c) Cox regression analysis exploring the factors associated with time to renal death

(a)		
Variables	F	p value
Diagnosis of gout	1.55	0.6
Allopurinol prescription	4.3	0.8
Entry serum urate level (bivariate)	0.14	< 0.05
Dyslipidaemia	0.01	< 0.05
Age	0.94	0.6
Entry eGFR	1.65	< 0.05
Hypertension	0.01	0.5
Diabetes	0.14	< 0.05
Body mass index	1.31	0.1

(b)		
Variables	t	p value
Diagnosis of gout	0.042	0.9
Allopurinol prescription	0.04	0.9
Entry serum urate level (bivariate)	1.68	< 0.05
Dyslipidaemia	1.5	0.1
Age	2.06	< 0.05
Entry eGFR	2.07	< 0.05
Hypertension	0.41	0.7
Diabetes	2.5	< 0.05
Body mass index	0.28	0.8

(c)				
	B	SE	Sig	Exp(B)
Age	− 0.023	0.004	< 0.05	0.98
Diabetes	− 0.109	0.143	0.45	0.9
Body mass index	− 0.006	0.009	0.52	1.0
Dyslipidaemia	− 0.146	0.139	0.3	0.86
Allopurinol	0.239	0.185	0.2	1.27
Entry eGFR	− 0.079	0.006	< 0.05	0.92
Entry serum urate level (bivariate)	− 0.015	0.167	< 0.05	0.86
Diagnosis of gout	− 0.044	0.004	0.81	0.96
Hypertension	− 0.299	0.183	0.1	0.74

Multivariate analysis was performed controlling for age, diagnosis of gout and prescription of allopurinol. This analysis showed a significant correlation between initial urate levels and delta eGFR. Additionally, a more extensive multivariate analysis involving comorbidities such as cardiovascular risk factors, initial eGFR and age still revealed a significant association between initial serum uric acid levels and delta eGFR (Table 2b).

### Association between diagnosis of gout and progression of kidney disease

There were 210 patients with a diagnosis of gout. Baseline characteristics between patients with and without a diagnosis of gout are summarised in Table 1b. Patients with a diagnosis of gout were significantly older with worse kidney function on entry to the registry. Furthermore, in patients with a diagnosis of gout, there was an increased prevalence of hypertension, diabetes mellitus and patients prescribed allopurinol treatment compared to patients without a diagnosis of gout. The prevalence of gout increased progressively until CKD stage 3 (Fig. 1b). There was no significant association between diagnosis of gout and delta eGFR on bivariate analysis with a mean of 2.2 (std dev: 6.7) in patients with no diagnosis of gout compared to 2.5 (std dev: 8.5) in patients with a diagnosis of gout (p = 0.6).

### Association between prescription of allopurinol and progression of kidney disease

**Fig. 2:**
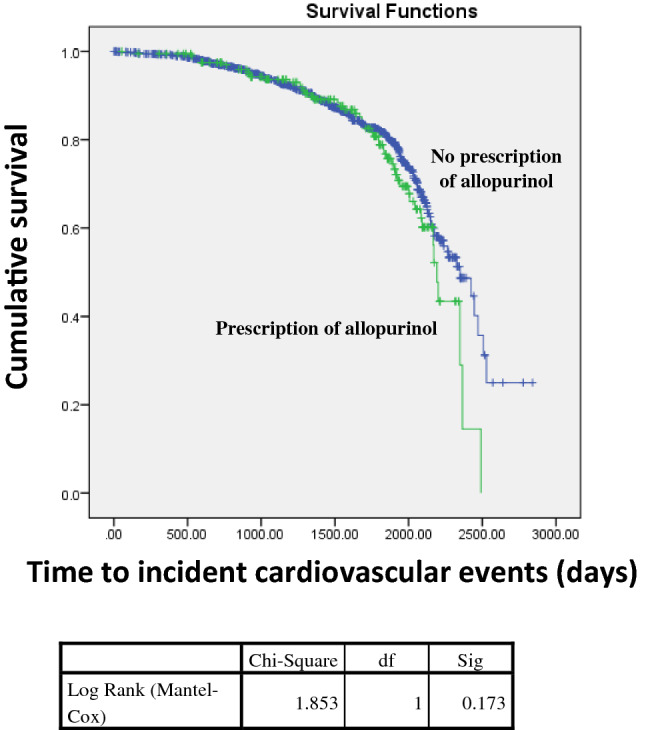
Kaplan Meier analysis exploring the association between allopurinol prescription and time to cardiovascular events

Two hundred and seven patients were prescribed allopurinol and within this group 21 (10.1%) commenced KRT during the study period and 59 (28.5%) died (Table 1c). By comparison, amongst those not prescribed allopurinol, 105 (11.5%) commenced KRT and 224 (24.5%) died. The proportion of patients prescribed allopurinol by CKD stage at baseline was 1.5% for stage 1, 7.1% for stage 2, 21.7% for stage 3, 21.4% for stage 4 and 17.3% for those in stage 5 (Fig. 1c). Patients who were prescribed allopurinol were older than those not prescribed allopurinol (70.7 years vs 65.8 years; p < 0.01), had a higher BMI (32.3 kg/m^2^ vs 30.5 kg/m^2^; p < 0.01), worse kidney function at time of consent (35.2 ml/min/1.73m^2^ vs 43.6 ml/min/1.73m^2^; p < 0.05), higher serum uric acid levels (0.5 mmol/L vs 0.4 mmol/L; p < 0.05) and higher proportions of diabetes mellitus (p = 0.04), dyslipidaemia (p < 0.01) and hypertension (p < 0.05); (Table 1c). On bivariate analysis, there was no significant difference in delta eGFR in patients that were prescribed allopurinol compared to those who were not prescribed allopurinol. The delta eGFR was 2.2 ml/min/1.73m^2^/year (std dev: 6.9) compared to 2.4 ml/min/1.73m^2^/year (std dev: 7.6) in patients who were prescribed allopurinol (p = 0.8). On multivariant analysis, age, diagnosis of gout and allopurinol prescription were not associated with delta eGFR (Table 2b). We also explored the association between allopurinol on new cardiovascular events that occurred during the follow up period in patients with CKD. Bivariate analysis revealed that there was no significant association between allopurinol prescription and cardiovascular events (p = 0.1). Moreover, Kaplan Meier analysis (Fig. 2) showed that there was no statistically significant difference in the time to cardiovascular events in patients with prescription of allopurinol compared to those without prescription of allopurinol (2053 days compared to 2216 days) (Log Rank p = 0.2).

### Association between hyperuricaemia, prescription of allopurinol and diagnosis of gout on the rate of progression to renal death and mortality

**Fig. 3:**
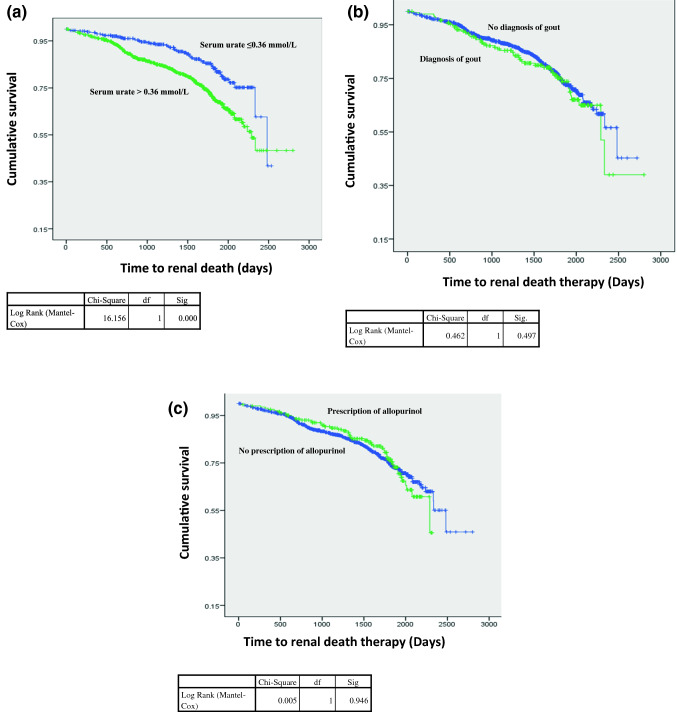
**a** Kaplan Meier exploring the time to renal death and initial serum uric acid level. **b** Kaplan Meier exploring the time to renal death and diagnosis of gout. **c** Kaplan Meier exploring the time to renal death and prescription of allopurinol

Kaplan Meier analysis revealed that hyperuricaemia was associated with a significantly shorter time to renal death. The mean time to renal death in patients with serum uric acid levels ≤ 0.36 mmol/L was 2225.1 days compared to 2148.1 days in patients with serum uric acid levels > 0.36 mmol/L. This was significant when tested by log rank (p < 0.05) (Fig. 3a). Cox regression analysis revealed patients with serum uric acid levels > 0.36 mmol/L were 1.9 times more likely to progress to renal death (p < 0.05). The mean age of patients without hyperuricaemia progressing to renal death was 60.9 years compared to 57.5 years in patients with hyperuricaemia (p = 0.3). The prescription of allopurinol was not associated with a significant change in the time to renal death, with mean days of 1980.6 in patients with allopurinol compared to 2206.1 in patients without allopurinol (log rank p = 0.9) (Fig. 3b). Cox regression analysis revealed that the hazard ratio between allopurinol and progression to renal death was 1 (p = 0.9). The mean age of patients without prescription of allopurinol progressing to renal death was 57.2 years compared to 62.6 years in patients with a prescription of allopurinol (p = 0.1). There was no significant difference in the time to renal death in patients with a diagnosis of gout when using log rank, with mean days of 2177.2 in patients without a diagnosis of gout compared to 2137.4 days in patients with a diagnosis of gout (p = 0.5) (Fig. 3c). The progression to renal death with a diagnosis of gout was 1.1 times greater than without a diagnosis of gout. However, this was not a significant result (p = 0.5). The mean age of patients without a diagnosis of gout progressing to renal death was 57.6 years compared to 60.2 years in patients with a diagnosis of gout (p = 0.4).

Kaplan Meier analysis, looking at patient deaths in the cohort, revealed that patients with serum urate  ≤ 0.36 mmol/L had significant difference in the time to death compared to patients with serum urate level > 0.36 mmol/L when using log rank, with mean days of 2958 compared to 2571 days, respectively (p < 0.05). There was no significant difference in the time to death in patients with prescription of allopurinol, with mean days of 2788 compared to 2590 (log rank p = 0.3). Moreover, there was no significant difference in the time to death in patients with a diagnosis of gout, with mean days of 2761 in patients without diagnosis of gout compared to 2606 days in patients with a diagnosis of gout (log rank p = 0.3).

## Discussion

In this study, there was an association between the rate of progression of CKD, including subsequent renal death, and serum uric acid levels. There was no association between the diagnosis of gout or prescription of allopurinol and progression to renal death. The presence of hyperuricaemia on CKD.QLD registry entry was associated with faster progression to renal death. However, it is important to note that the patients with hyperuricaemia had worse baseline kidney function as well as a higher incidence of cardiovascular risk factors such as dyslipidaemia and diabetes, which could have contributed to faster CKD progression. Importantly, on multivariate analysis there was still an association between serum uric acid levels and renal outcomes after adjusting for the diagnosis of gout, prescription of allopurinol, age and entry eGFR.

At present, the literature on the effect of serum uric acid levels, allopurinol prescription and the diagnosis of gout on renal outcomes is inconclusive. Some studies reported that raised serum uric acid levels accelerated progression of CKD [16]. However, this has not been consistent across all reported CKD cohorts in the literature. Declining kidney function is associated with rising serum uric acid levels due to decreased excretion, thus confounding the analysis of the effect of high serum uric acid levels on the progression of kidney disease. It is difficult to evaluate the independent role of serum uric acid as a causative agent rather than a secondary result on the risk of kidney function decline and incidence of ESKD amongst patients with CKD.

In our study, prescription of allopurinol was not associated with worsening renal outcomes. We also analysed the impact of allopurinol in patients with hyperuricaemia. However, even after focusing on allopurinol use in patients with hyperuricaemia, we could not identify an association between allopurinol prescription and renal outcomes. Others report similar findings. Siu and colleagues [14] used a randomised control design to identify the association between allopurinol and renal outcomes. In this small study of 54 patients followed up for 12 months, kidney function did not have a statistically significant decline (defined by > 40% increase in serum creatinine) in the cohort receiving allopurinol [14]. In contrast, Goicoechea and colleagues [13] reported a significant, positive association between renal outcomes (defined by delta eGFR) and allopurinol in a small randomised control study. The study followed patients for a longer time of 24 months [13]. A metanalysis performed by Kang and Colleagues [17] found an overall statistically significant benefit with the use of allopurinol in CKD with a general trend towards improvement in renal outcomes. The CKD-Fix Trial and the Perl Trial were recent randomised controlled trials exploring the effect of allopurinol on renal outcomes. However, both these studies failed to reveal any relationship between prescription of allopurinol on progression of kidney function in a CKD setting [18, 19]. Moreover, given the frequent use of allopurinol in CKD patients, as well as its hypothesised effect on vasculature, we also explored the association of allopurinol prescription on new incident cardiovascular events. However, neither the chi square analysis nor the Kaplan Meier analysis showed any significant associations between these two factors. Furthermore, there was no significant association between allopurinol prescription and death in the CKD cohort.

The third relationship we explored was the effect of the diagnosis of gout on progression of kidney disease. We hypothesised that given the occurrence of clinical manifestations from hyperuricaemia, diagnosis of gout may be associated with faster progression of CKD. Our results did not support this hypothesis. There was no significant change in the renal outcomes. There was also no significant association with time to renal death in patients with diagnosis of gout in this CKD cohort. Interestingly, the literature looking at this issue is scarce. Most of the literature has been focused on exploring the prevalence of CKD in patients with a diagnosis of gout, with no studies exploring the rate of kidney deterioration.

The strengths of our work include the use of delta eGFR to quantify the effect of the three interlinked variables on the progression of kidney dysfunction in CKD. This allowed a quantitative design of evaluation. Moreover, there was a relatively large number of patients included in the study. Furthermore, incorporating the diagnosis of gout, hyperuricaemia, and prescription of allopurinol to analyse the renal outcomes reduces loss of the sentient uric acid variables. However, there were several limitations to this study. Firstly, this study was a single centre study and this limits the generalisability of our findings. It would be hard to compare the findings of our study with other centres containing different environmental and socioeconomic profiles. Secondly, we lacked data on adherence patterns. This is especially important in evaluating the association between allopurinol prescription and kidney function deterioration. Thirdly, the threat of residual confounding factors cannot be excluded in a non-randomised study using registry-based retrospective data and this could explain some of the discrepancies in our results compared to other studies. Fourthly, the delta eGFR values were low with a high standard deviation. This could be due to the number of patients whose renal function improved over the course of the follow up period which could have affected our results.

In summary, we undertook a retrospective cohort study investigating the relationships between hyperuricaemia, allopurinol prescription, diagnosis of gout and renal outcomes We observed an increased prevalence of hyperuricaemia and gout with more advanced stages of CKD. Moreover, we identified an association between hyperuricaemia and faster CKD progression, as well as reduced time to renal death. However, we did not observe an association between prescription of allopurinol, diagnosis of gout and renal outcomes Moreover, prescription of allopurinol was not associated with decreased incident cardiovascular events in the CKD cohort. These data suggest that the hyperuricaemia is likely the effect and not the cause of CKD. Randomised control trial evidence with a longer follow up remains the gold standard to clarify the effect of allopurinol and uric acid lowering therapies on renal outcomes in a CKD cohort.
